# High-throughput development of simple sequence repeat markers for genetic diversity research in *Crambe abyssinica*

**DOI:** 10.1186/s12870-016-0828-y

**Published:** 2016-06-18

**Authors:** Weicong Qi, Feng Lin, Yuhe Liu, Bangquan Huang, Jihua Cheng, Wei Zhang, Han Zhao

**Affiliations:** Institute of Biotechnology, Provincial Key Laboratory of Agrobiology, Jiangsu Academy of Agricultural Sciences, Nanjing, 210014 China; Department of Crop Sciences, University of Illinois, Urbana-Champaign, IL 61801 USA; College of Life Science, Hubei University, Wuhan, 430062 China; Waksman Institute of Microbiology, Rutgers University, 190 Frelinghuysen Road, Piscataway, NJ 08854 USA

**Keywords:** *Crambe abyssinica*, EST-SSR, SSR, Molecular breeding, Genetic diversity, Next-generation sequencing, *de novo* assembly

## Abstract

**Background:**

The allohexaploid *Crambe abyssinica* (crambe) is an oilseed crop that has been recognized for its potential value in the chemical industry, particularly in terms of producing high-erucic acid content vegetable oil. However, as an understudied crop, improvement of crambe has been hampered by the lack of genetic and genomic information to enhance its yield, oil quality and resistance against biotic and abiotic stress. Development of molecular markers is therefore of great significance to facilitate genetic improvement of crambe.

**Results:**

In this study, high-throughput sequencing was performed to generate sequences for the transcriptome and genome of a widely planted crambe cultivar, Galactica. A total of 186,778 expressed sequence tag (EST) contigs as 8,130,350 genomic contigs were assembled as well. Altogether, 82,523 pairs of primers were designed in the flanking sequences of the simple sequence repeat (SSR) within these contigs. Virtual PCR analysis showed that a fraction of these primers could be mapped onto the genomes of related species of *Brassica*, including *Brassica rapa*, *B. oleraceae* and *B. napus*. Genetic diversity analysis using a subset of 166 markers on 30 independent *C. abyssinica* accessions exhibited that 1) 95 % of the designed SSRs were polymorphic among these accessions; 2) the polymorphism information content (PIC) value of the markers ranged from 0.13 to 0.89; 3) the genetic distances (coefficient NEI72) between accessions varied from 0.06 to 0.36. Cluster analysis subsequent on the accessions demonstrated consistency with crambe breeding history. F-statistics analysis revealed a moderate level of genetic differentiation in *C. abyssinica* (Gst = 0.3934) and a accordingly low estimated gene flow (Nm = 0.7709).

**Conclusion:**

Application of high-throughput sequencing technology has facilitated SSR marker development, which was successfully employed in evaluating genetic diversity of *C. abyssinica* as demonstrated in our study. Results showed these molecular markers were robust and provided powerful tools for assessing genetic diversity and estimating crambe breeding history. Moreover, the SSR primers and sequence information developed in the study are freely available to the research community.

**Electronic supplementary material:**

The online version of this article (doi:10.1186/s12870-016-0828-y) contains supplementary material, which is available to authorized users.

## Background

*Crambe abyssinica* (crambe) is an allohexaploid (2n = 6× = 90) with an estimated genome size of approximately 3.5 Gb based on its 2C-value (=7.04 pg) [1–5]. A member of the genus *Crambe abyssinica* distribute unevenly among four major geographical regions: Macronesian, Mediterranean, East Africa, and Euro-Siberian-southwest Asia [6]. *C. abyssinica* originated from the Mediterranean region and has been cultivated mainly for producing high-erucic acid plant oil, a natural product of interest to the chemical industry [2, 3]. Its breeding and cultivation first started in Europe from the 1900s, and then subsequently spread throughout the world [7]. Previous efforts in improving crambe’s agronomic traits included traditional breeding [7], mutagenesis, and transgenesis [7–11]. Despite obvious improvement on agronomic traits, genetic and genomic information on this oilseed crop is still largely limited. So far, only a few hundred nucleotide sequences of DNA and RNA in *C. abyssinica* were deposited in public database (National Center for Biotechnology Information), markedly incomparable to other oil crops, e.g. *Brassica napus*, soy bean, peanut or sunflower. The paucity of available information on nucleotide sequences has hindered its genetic studies, such as molecular marker development, linkage map construction and gene discovery.

The next-generation sequencing (NGS) technologies have significantly increased the speed and throughput of sequence information acquisition, and greatly accelerated the discovery process for molecular markers *e. g*. single nucleotide polymorphisms (SNP) and SSR [12, 13]. Although SNP markers are popular and widely applied in major crops, SNP detection usually requires expensive chemistries and equipment which limited its application. SSR markers are technologically less demanding and have advantages including high level of polymorphism, low cost, reproducibility and transferability across species. For example, a total of 82 barley EST-derived SSR primer pairs were tested for transferability to H. *chilense*, all of which amplified products of correct size from this species [12]. Moreover, in some species, SSRs were found to be more informative than SNPs, for instance Singh et al. [13] compared the use of SSR and SNP markers in estimation of genetic diversity and population structure of Indian rice varieties, and concluded that SSR were more efficient for diversity analysis.

In the study, we have employed the NGS sequencing technology to sequence genomic DNA and EST of *Crambe abyssinica* with the goals of characterizing simple satellite repeat loci and developing corresponding markers. Our study demonstrated the procedures for developing SSR markers for *C. abyssinica*, facilitated by the use of the next-generation sequence technology. The developed SSR markers were utilized to evaluate the genetic diversity of crambe accessions, which exhibit a moderate level of genetic differentiation in *C. abyssinica* and a correspondingly estimated gene flow. The nucleotide sequences generated in present study including the SSR-primers and the assembled genomic and transcriptomic contigs are freely available to the research community, and will serve as useful genetic resource for this species.

## Results

### *De novo* assembly of transcriptomic and genomic DNA of *Crambe abyssinica*

**Fig. 1:**
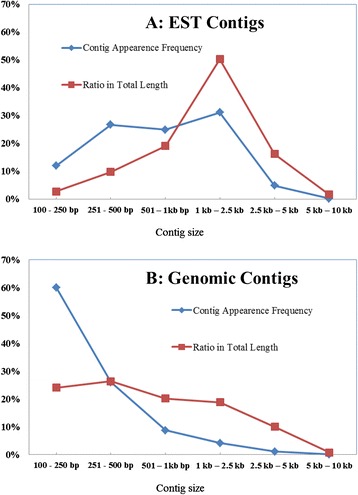
The distribution of the assembled contigs in different sizes. Chart A and B has showed the size distribution of the assemble EST and genomic contigs respectively. The line in blue indicated the appearance frequencies of the contigs in different size, and the red line showed the corresponding ratio in total length

The total RNA of developing crambe seeds (21 days after pollination) was isolated and synthesized cDNA was sequenced by Illumina Pair-End 100 × 2, from which a total of 4.0 Gb sequence data were generated. The deduced coverage of the transcriptome was about 20×. The raw data were processed using Q20 quality control and L40 length filtering. The remaining raw reads were assembled using the Trinity program to generate contigs. A total of 234,622 contigs were generated from the *de novo* assembly of cDNA. Then, the contigs were filtered using BLASTN (1E-50) to remove those with high similarity. Finally 186,778 contigs (209 Mb) remained for EST-SSR calling. The lengths of the contigs varied from 100 bp to > 10 kb with the average 1,138 bp (N50 = 1,428 bp). The genomic DNA isolated from fresh leaves was sequenced by Illumina similar to that of the cDNA. A total of 33.5 Gb raw data was obtained, indicating a 9.5× depth of coverage. The raw data were processed with EST data, except for being assembled by a ‘Short Oligonucleotide Analysis Package program *de novo*’ (SOAPdenovo). Finally, 8,130,350 contigs (1.41 Gb) were assembled for genome-SSR calling (Table 1). The average size of the contigs was 186 bp, and N50 is 275 bp with the longest sequence length of 8,770 bp. Figure 1 showed the distribution of the assembled EST and genomic contigs according to their lengths. The EST contigs between 1 to 2.5 kb were the most abundant. Among the genomic contigs, those within 0.1 to 0.25 kb were the most prevail in terms of contig appearance frequency, but the contigs within 0.25 to 0.5 kb were the most dominant in terms of the ratio in total length.

**Table 1:** Summary of the *de novo* assembly of transcriptome and genome and SSR loci calling

	Transcriptome	Genome
Raw data	4.0 Gb	33.5 Gb
Total number of contigs	186,778	8,130,350
Total length of contigs	209 Mb	1.41 Gb
Total number of the contigs with SSR locus	19,674	89,983
The frequency of SSR occurrence	One locus per 11.1 kb	One locus per 16.8 kb
Contigs N50	1,428 bp	275 bp
Average contig length	1,138 bp	186 bp
Maximum contig length	16,475 bp	8,770 bp

### SSR development, primer design and preliminary selection

**Fig. 2:**
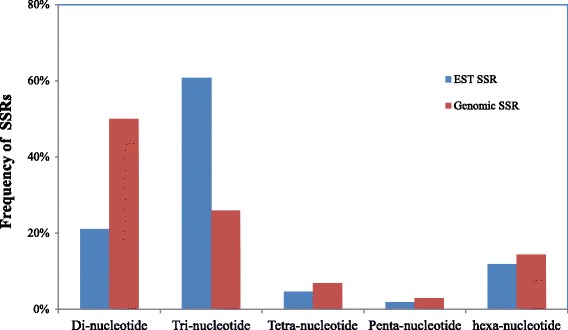
SSRs Statistics. The frequency distribution of the SSR motif was showed in the chart. The nucleotide repeat motif varied from di-nucleotide to hexa-nucleotide as showed by the X axis. The tri- and di-nucleotide were the most prevail in the EST and genomic SSR respectively

**Fig. 3:**
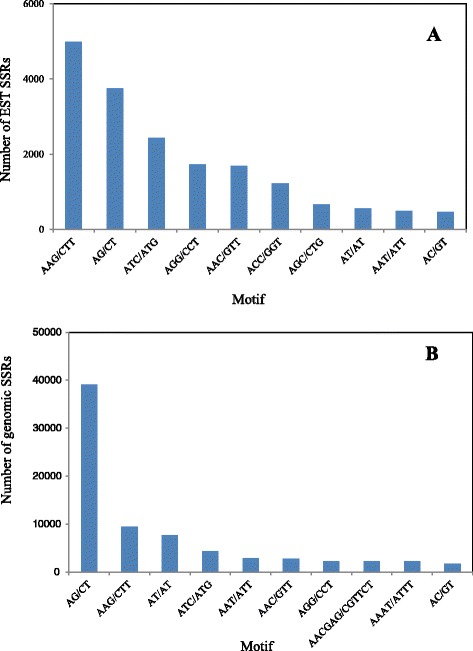
Distribution of top-10 motifs in EST-SSRs (**a**) and genomic SSRs (**b**). The most common EST-SSRs were trinucleotide motif AAG/CTT, whereas the predominant genomic SSRs were dinucleotide motif AG/CT. The X-axis represents the motif sequence, and the Y-axis represents the number of detected SSRs

SSR loci were called using the MISA program from the assembled transcriptomic and genomic contigs. The parameters were designed for identifying perfect di-, tri-, tetra-, penta-, and hexanucleotide motifs with a minimum of 8, 5, 4, 4, and 3 repeats, respectively. As showed in Table 1, among the *de novo* assembled contigs, 19,674 cDNA contigs out of 186,778 (10.5 %), and 89,983 genomic contigs out of 8,130,350 (1.1 %) contained at least one SSR locus. On average, there was one SSR locus in every 11.1 kb cDNA and 16.8 kb genomic DNA, respectively. Eventually, a total of 22,734 EST derived SSR loci and 97,170 genomic SSRs loci were detected. A total of 274 EST-SSR motifs and 455 genomic SSR motifs were identified. Of the EST-SSR motifs, there were 3 di-, 10 tri-, 24 tetra-, 38 penta and 199 hexa-nuleotide; and of the genomic SSR motifs, there were 3 di-, 10 tri-, 31 tetra-, 89 penta and 322 hexa-nuleotide. The Fig. 2 demonstrated the statistics in the SSR loci detected. Of the EST-SSR loci, trinucleotides accounted for 60 % and thus ranked the most abundant, followed by dinucleotides (21 %), hexanucleotides (11 %), tetranucleotides (5 %), and pentanucleotides (2 %) (Fig. 2). While for the detected genomic-SSR loci, 50 % were dinucleotide motifs, followed by 26 % trinucleotides, 14 % hexanucleotides, 7 % tetranucleotides and 3 % pentanucleotide (Fig. 2). The top ten most abundant nucleotide repeat types in the newly detected SSRs were presented in Fig. 3, with the most common EST-SSR motif being AAG/CTT and the most abundant genomic-SSR motif being AG/CT.

Primer pairs with melting temperatures (T_m_) within the range of 55 °C to 61 °C and with amplicons within a size range of 150 bp to 400 bp were designed from the flanking sequences of the SSR loci by using the Primer 3 program. Primers were then validated by *in silico* PCR against the crambe genome contigs per se. Those primer sets amplifying multiple target regions were discarded to ensure the specific locations of the primers. Finally, a total of 3,803 pairs of EST-SSR primers (Additional file 1) and 78,720 genomic-SSR primer pairs (Additional file 2) were obtained and the ratio between EST- and genomic-SSR primer pairs was around 1:20.

### Validating the primers with the whole-genome data of *Brassicaeace* crops

**Fig. 4:**
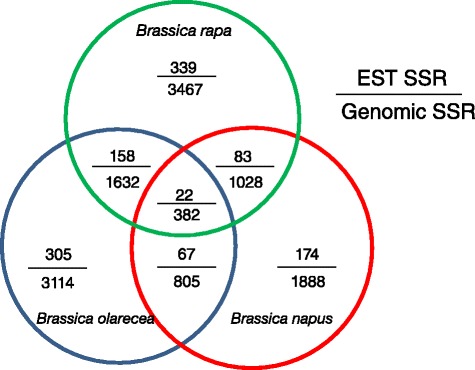
The number of the primers mapped on genomes of *Brassica* species. In the chart the numbers of the new primer-pairs which were mapped on *B. rapa*, *B. oleracea* and *B. napus* were showed as well as the number of the overlapped primer-pairs

**Fig. 5:**
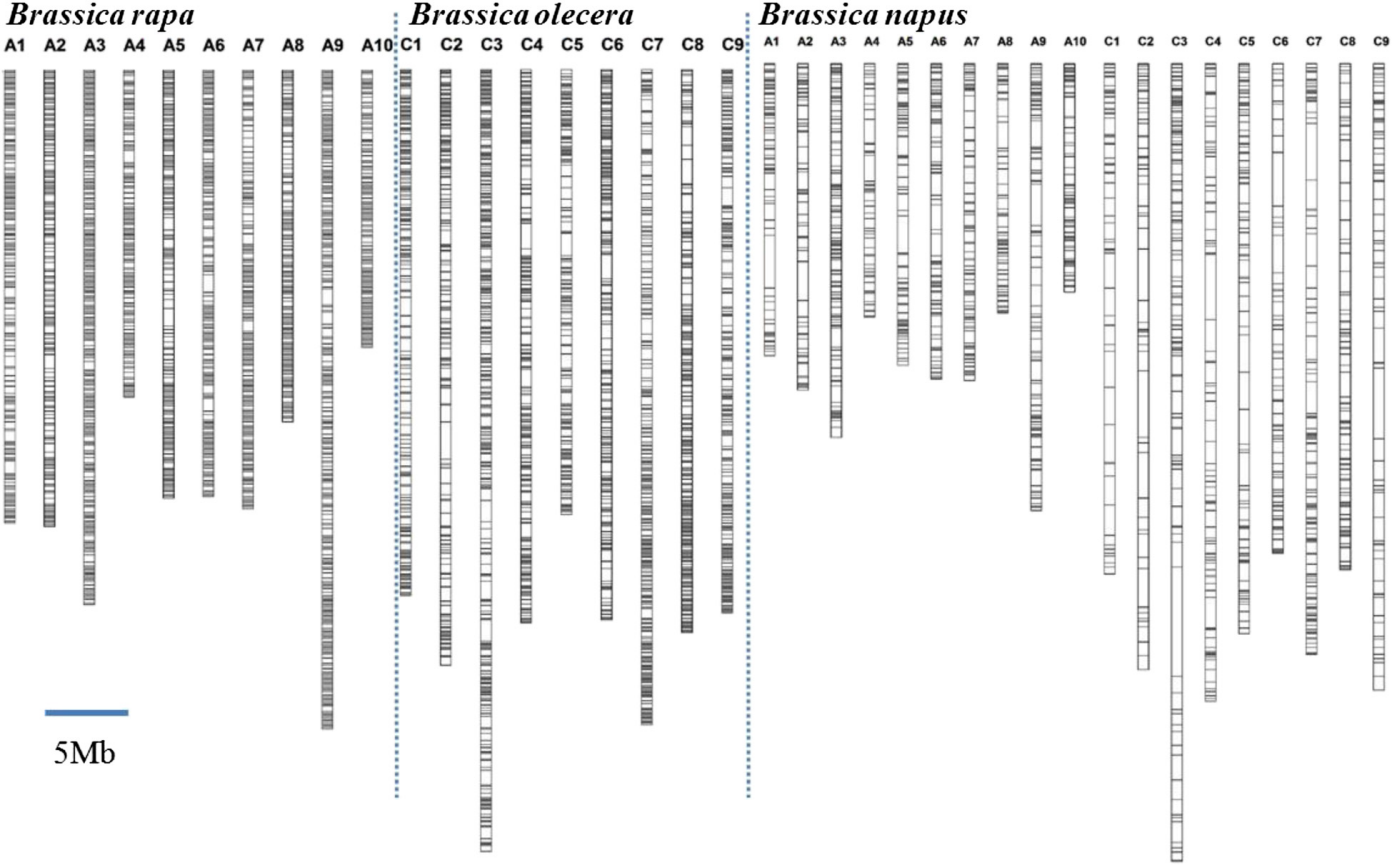
Distribution of *C. abyssinica* EST- and genomic-SSRs in the genomes of *Brassica rapa*, *Brassica olarecea* and *Brassica napus*. A total of 5,718 loci derived from EST- and genomic-SSR markers were mapped onto chromosomes of *Brassica rapa, Brassica olarecea* and *Brassica napus*, distributed at a rate of one marker per 10 kb

*Brassica rapa*, *Brassica olecera*, and *Brassica napus* belong to the same family *Brassicaceace as* crambe. To assess the intergeneric homology of the newly developed crambe SSRs across *Brassicaceace*, the EST- and genomic-SSR primers were tested by *in silico* PCR against the genome of *B. rapa*, *B. oelcera* and *B. napus*. The maximum number of mismatches at the 5′ end is three base pairs, with none allowed at the 3′ end. The primer pairs that amplified a single band between 200 bp and 500 bp were considered as the preferred molecular markers. Finally, 339 pairs of EST-SSR primers and 3,467 pairs of genomic-SSR primers were mapped onto *B. rapa* genome (Additional file 3), 305 and 3,114 on *B. olarecea* (Additional file 4), 174 and 1,888 on *B. napus* (Additional file 5). The overlapped primers among three genomes were showed in Fig. 4, where 22 EST-SSR primer pairs and 382 genomic-SSR primer pairs overlapped across all three genomes, suggesting the corresponding amplified loci are evolutionarily conserved in the family. According to the physical location, these primer pairs distributed at a frequency of one marker per 10 kb in *Brassica rapa* and *Brassica olecera* (Fig. 5).

### Evaluating the diversity of the *C. abyssinica* germplasm collections by using the newly developed SSR markers

A total of 166 primer pairs (Additional file 6) consisting of 13 EST-SSRs and 153 genomic-SSRs were selected and utilized in the PCR analysis of genomic DNA from 30 crambe accessions to validate the utility and reliability of these SSR markers in *C. abyssinica* germplasm identification (Table 2). In terms of geographic origins, the accessions can be classified into four groups: the Mediterranean group (accessions from Ethiopia, Spain, and Turkey), the Middle European group (accessions from Ukraine, Former Soviet Union, and Romania), the Western European group (accessions from German and Holland), and the USA group. Amplified products were analyzed by polyacrylamide gel electrophoresis.

**Table 2:** Summary of the PCR analysis

PI#	Origination	Name	Group	TNA	SNA	TTNA	GNA	PGNA
281737	Ukraine		Eastern Europe	569	2	858	273	0
306422	Romania			609	6			1
393513	FSU			609	0			2
393514	FSU			613	1			2
393515	FSU			603	0			1
633197	Germany	CR 1699	Western Europe	564	2			3
Galactica	Holland	Galactica		464	12			13
304399	Denmark		Northern Europe	589	1			4
304400	Denmark			613	5			1
360889	Sweden			627	4			2
360890	Sweden			644	4			2
360891	Sweden			639	1			1
360892	Sweden			651	1			2
360893	Sweden			615	1			2
392071	Spain		Mediterranean	625	2			2
392072	Spain			629	2			2
392326	Turkey			629	6			4
392327	Turkey			629	2			2
384531	Ethiopia	Ames 1433		606	2			2
384532	Ethiopia	Ames 1434		624	4			2
384533	Ethiopia	Ames 1435		623	2			2
414156	US, Iowa	Ames 1657	USA	589	1			1
514649	US, Indiana	Meyer		562	3			1
514650	US, Indiana	Prophet		588	1			2
533664	US, Maryland	C-22		598	2			1
533665	US, Maryland	C-29		580	1			1
533666	US, Maryland	C-37		575	5			1
533667	US, Maryland	BELANN		552	1			4
533668	US, Maryland	BELENZIAN		587	3			7
633196	US, New Mexico	NM 85		557	0			3

*Note*: The accessions with PI numbers are those from US Department of Agriculture. *Abbreviation*: *TNA* tested number of alleles, *SNA* specific number of alleles, *TTNA* total number of alleles, *GNA* general number of alleles, *PGNA* primers giving not generate alleles

Results revealed that the tested EST- and genomic-SSR markers were highly polymorphic among the accessions. The polymorphism information content (PIC) value of the primers varied from 0.13 to 0.89 (Additional file 6). There were nine primer pairs showing no polymorphisms among the accessions. As shown in Table 2, a total of 858 alleles were detected in 30 accessions, wherein 77 alleles were determined to be accession-specific and 273 alleles were generally detected in all the accessions.

**Fig. 6:**
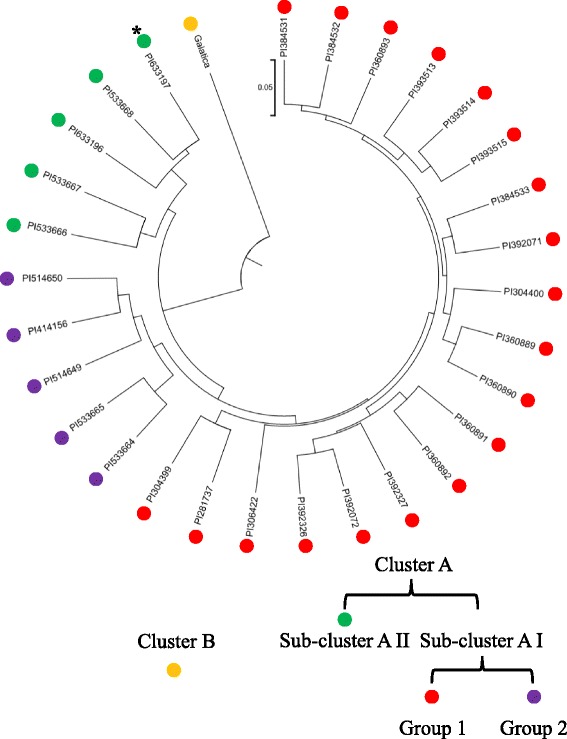
Tree-plot derived from UPGMA cluster analysis using NEI72 coefficient of SSR markers. The UPGMA (unweighted pair group method analysis) tree-plot was generated using Mega 6.0 cluster analysis based on the genetic distances (calculated with coefficient of NEI72 by NTSYS software) of 30 independent accessions. The names and geographic originations of the accessions were listed in Table 2. The asterisk marks accession PI633197 from Germany

The genetic distances with the coefficient of NEI72 among accessions ranged from 0.06 to 0.36 (Additional file 7). The largest genetic distance was observed between Galactica and PI306422 from Romania. Unweighted pair group method analysis (UPGMA) was used to cluster the accessions, which indicated that the 30 accessions could be grouped accordingly into two clusters (Fig. 6). Cluster A included all the accessions except Galactica and could be further subdivided into two sub-clusters. Sub-cluster AI contained two groups: Group 1 consisted of the accessions from the origin of *C. abyssinica* (Mediterranean region) and surrounding areas, whereas Group 2 comprised one from Iowa, US (PI414156), two from Indiana (‘Prophet’/PI514650; ‘Meyer’/PI514649) and US, two accessions (‘C-22’/PI533664; C-29/PI533665) from Maryland, US. Sub-cluster AII included three accessions from Maryland (‘BelEnzian’/PI533668; ‘BelAnn’/PI533667; ‘C-37’/), US and one (PI414156) from New Mexico, US and one (PI633197) from Germany. In comparison with other American accessions, the accessions collected from Iowa and Indiana had a closer relationship with the ones from Mediterranean region and surrounding areas.

F-statistics analysis of the PCR results showed a generally moderate level of genetic differentiation among the accessions (Gst = 0.3934), and a corresponding low estimated gene flow (Nm = 0.7709). The accessions from the Mediterranean region and surrounding areas (Europe, Africa, and Asia) showed a similar degree of genetic differentiation (Gst = 0.3866, corresponding to an estimated gene flow Nm = 0.7934). In addition, the accessions from the USA showed a relatively lower level of genetic differentiation and a higher estimated gene flow (Gst = 0.1868, corresponding estimated gene flow Nm = 2.1764).

## Discussion

In present study, SSR markers for the hexoploid species *Crambe abyssinica* from Mediterranean region were developed based on *de novo* assembled cDNA and genomic DNA from cultivar Galactica released by Wageningen University, the Netherlands. The reliability of these newly developed SSR primers was tested via PCR analysis on a total of 30 different crambe germplasm accessions, including the modern cultivars ‘Prophet’ (PI514650), ‘Meyer’ (PI514649), ‘BelEnzian’ (PI533668), ‘BelAnn’ (PI533667), ‘C-22’ (PI PI533664), ‘C-29’ (PI533665), and ‘C-37’ (PI533666) from the USA and Galactica from the Netherlands. The UPGMA tree plot in Fig. 6 showed a reasonable and conclusive topology that was consistent with the described breeding history of *C. abyssinica* [14]. For example, the figure showed ‘Prophet’ and ‘Meyer’ had closer relationship with the main accessions from Mediterranean and neighboring areas than ‘C22’, ‘C29’, ‘C37’, BelEnzian and BelAnn. It automatically reflected the fact that ‘Prophet’ and ‘Meyer’ were obtained from mass selection and crossing in two initial accessions from Sweden and Ethiopia; and ‘C22’, ‘C29’, ‘C37’, BelEnzian and BelAnn also originated from these two initial accessions but with extra breeding process and longer term of selection [15]. The figure also showed that American accessions were rather isolated from those from European, Asian, and African accessions. It indicated that although crambe breeding in USA was initiated in the1960s from the germplasms of European origin, the breeding effort since then was rather intensive and relatively independent. German accession PI1633197 was an exception which showed a close relationship with the accession from New Mexico, USA. The information regarding this accession is limited in that it was collected by the Institut fur Pflanzengenetik und Kulturpflanzenforschung, Germany, and later donated to United States Agricultural Department, whereas its place origin was unclear. Based on the estimated genetic distances, we deduced hypothesized that: 1) PI1633197 derived from late-breeding accessions; 2) it originated from a germplasm of the USA. Galactica was also a European cultivar which is known as the latest cultivar. It shared its genetic background with all the accessions from USA, Europe, and Mediterranean region, because it was bred from a European landrace and a late American line [15]. F-statistics analysis based on PCR results indicated a moderate level of genetic differentiation among the 30 accessions examined (Gst = 0.3934), and gene flow was low (Nm = 0.7709). Geographical isolation, as well as artificial breeding and selection, might have likely caused the genetic differentiation. The genetic differentiation within the USA accessions (Gst = 0.1868) was quite lower than that of the accessions from the Mediterranean and surrounding areas (Europe, Ethiopia, and Turkey, Gst = 0.3866). These findings indicated that: 1) The cultivation history of *C. abyssnica* in the USA was relatively shorter than that in the Mediterranean and surrounding area; and 2) The crambe genetic resource in the USA was less abundant. The genetic differentiation approach based on the polymorphisms in the newly developed SSRs also demonstrated that those markers for *C. abyssnica* and the corresponding primers were reliable and robust.

Among the *de novo* assembled contigs, it was found that 10.5 % EST-contigs (19,674 out of 186,778) contained SSR loci, which was consistent with those of dicotyledonous plants, which ranged from 2.65 % to 16.82 % [16]. The frequency of genomic-SSR occurrence was one per 16.8 kb which was lower than what has been reported in *Brassica* species, for instance one locus every 2.5, 2.9 and 2.8 kb in the genome of *B. rapa*, *B. oleracea* and *B. napus* respectively [17]. It was mainly because that the mono-nucleotide repeat tandem was excluded in present research. The most abundant dinucleotide motif in crambe genome and EST was poly (AG/CT), which was the same as *Arabidopsis* genome and ESTs [18], ESTs of *Brassica rapa* subsp. *Pekinensis* [19], genome of *B. rapa* subsp *chinensis* [20], *Medicago truncatula* [21] and *Raphanus sativus* [22]. But in the genomes of *B. napus*, *B. rapa* subsp. *Pekinensis* and *B. oleracea*, poly (AT/TA) was the most abundant [17], when poly (AG/CT) ranks as the second [23]. In the EST or genome SSR of Arabidopsis and genus *Brassica*, the occurrence of poly (GC/CG) was rare, which was as same as what has been found here. Previous studies showed the trinucleotide tandem repeat occurred more frequently in coding region than in non-coding regions [24]. And in present research we also found that, relatively, there was more tri-nucleotide SSR in ESTs than in the genomic contigs. The most common triplet repeat detected in crambe EST and genome was poly (AAG/TTC), which was the same in Arabidopsis [18], *B. rapa* [19], *B. napus*, *B. olarecea* and many other plant species [24]. On the other hand, in *C. abyssinica*, the SSRs of tetra- and penta-nucleotide motifs were relatively rare when the hexa-nucleotide SSRs were abundant, in comparison with *Brassica* crops [17]. But as same as *Brassica* crops, the A/T rich motifs rich in for instance AAC, AAG, AAT, AAAC, AAAG, AAAT, AAAAC, AAAAG, AAAAT and so on were dominant [17]. The PIC values of the crambe SSR varied from 0.13 to 0.89, which was comparable to those of *Brassicaceae* species and other plant reported [20, 25]. Previous research reported the intergeneric transferability of plant SSRs, for example the SSR primers developed from Arabidopsis could be engaged in *Brassica* species (*B. napus*, *B. rapa, B. oleracea*, *B. nigera*, *B. juncea*, and *B. carinata*) and showed polymorphism [26]. In present research, the BLAST analysis also showed that there were a number of crambe SSRs could be mapped on the genomes of *B. rapa*, *B. oleracea* and *B. napus*. This suggested 1) the potential intergeneric transferability of the newly developed molecular markers in *Brassicaceae* family; 2) the whole-genome sequence of *Brassica* crops [27] or *Arabidopsis* [28] could serve as references for genomic and genetic research studies on other *Brassicaceae* species.

There were 166 pairs of primer selected for PCR validation where primers amplified single band in virtual PCR and evenly distributed throughout the *Brassica rapa* genome. When they were employed for the PCR validation, most of these SSR primers however generated multiple bands. In 30 independent accessions, a total of 858 alleles were detected, and an average of 5 alleles was tested using each primer pair. A total of 273 alleles were generally observed among all accessions, suggesting that these genomic sequences were conserved and duplicated in the crambe genome. Correspondingly, 77 alleles were found accession-specific. Compared to the other accessions, Galactica has the highest number of specific alleles, this finding was reasonable because the markers were designed based on its nucleotide sequences. Also as the latest cultivars, Galactica underwent a longer term of selection, the specific alleles probably correspond artificial selection effect. On the other hand, some primer pairs failed to generate any band patterns (as showed in Table 2) in certain accessions, but no primer pair was unable to generate any polymorphic bands across accessions. This observation may be attributable to various technical reasons such as the primers were designed to cover splice sites; a large intron was present, or the cultivars harbored presence/absence variation. In the future, more cultivars will be sequenced with large genetic distance to better elucidate SSR locus variation and effectively developed corresponding SSR markers.

## Conclusion

The present study has developed a large set of SSR markers for *C. abyssinica* using high-throughput transcriptome and genome sequencing technologies. 166 of the identified primer pairs were used in the PCR analysis on 30 different accessions. Results showed that: 1) 90 % of the primers generated polymorphic bands; 2) the PIC value of the primers ranged from 0.13 to 0.89; and 3) the genetic distances between accessions ranged from 0.06 to 0.36. Cluster analysis based on genetic distances demonstrated that the accessions could be classified into a manner that was consistent with crambe breeding history. F-statistics analysis of the PCR results showed that the genetic differentiation of *C. abyssinica* (Gst) was 0.3934, and its corresponding estimated gene flow (Nm) was 0.7709. These results suggested that due to geographical isolation and artificial selection, *C. abyssinica* has adapted moderate level of genetic differentiation and gene flow. SSR primers and sequence information developed in the present study are freely available to the research community, serving as a useful and robust resource for molecular taxonomy studies, linkage map construction, and molecular marker-assisted breeding.

## Methods

### Plant materials and isolation of DNA and RNA

*C. abyssinica* cultivar Galactica was used for genome and cDNA sequencing. Another 29 accessions (acquired from USDA germplasm resources) were used for SSR testing and cluster analysis. Crambe seeds were germinated in Petri dishes with two layers of fully wetted filter paper and kept at 25 °C in the dark. Upon radical emergence, the seedlings were transferred to soil and kept in a greenhouse with an average temperature of 20 °C.

Genomic DNA was isolated from young leaves of 30-day-old crambe plants after seed germination following the method described by Aldrich and Cullis (1993), but with 1 % (w/v) polyvinylpyrrolidone-10 in a DNA extraction buffer.

Total RNA of developing seeds was extracted from bulked seeds of T0 plants [(10 seeds per plant, 21 days after flowering (DAF)] using RNeasy Plant Mini Kits (Qiagen, Germany), following the manufacturer’s instructions. The isolated RNA was treated with RNase-free TURBO DNase (Ambion, USA) to remove residual genomic DNA. First-strand cDNA synthesis was conducted using 20-μL reaction mixtures containing 1 μg of total RNA with iScript™ cDNA Synthesis Kit (Bio-rad, USA).

### Illumina sequencing

Illumina sequencing was conducted at Berry Genomic Ltd., in Beijing, China, following the manufacturer’s instructions (Illumina, San Diego, CA). mRNA with a poly (A) tail was isolated from 20 μg of total RNA using Sera-mag magnetic oligo (dT) beads (Illumina). To avoid priming bias, the purified mRNA was first fragmented into small pieces (100–400 bp) using divalent cations at 94 °C for 5 min. Using random hexamer primers (Illumina), the double-stranded cDNA was synthesized using the SuperScript double-stranded cDNA synthesis kit (Invitrogen, CA). The synthesized cDNA was subjected to end-repair and phosphorylation, and then the repaired cDNA fragments were 3' adenylated by using Klenow Exo- (3′ to 5′ exo minus, Illumina). Illumina paired-end adapters were ligated to the ends of these 3′-adenylated cDNA fragments. To select the proper templates for downstream enrichment, the products of the ligation reaction were purified on 2 % agarose gel. The cDNA fragments (about 200 bp in size) were excised from the gel. Fifteen rounds of PCR amplification were conducted to enrich the purified cDNA template using PCR primers PE 1.0 and 2.0 (Illumina) using Phusion® DNA polymerase. Finally, the cDNA library was constructed using 200-bp insertion fragments. After validating on an Agilent Technologies 2100 Bio-analyzer, the library was sequenced on an Illumina HiSeq TM 2000 system (Illumina Inc., San Diego, CA, USA) using the following workflow: template hybridization, isothermal amplification, linearization, blocking, sequencing primer hybridization, and sequencing on the sequencer for read 1. After completion of the first read, the template scan was regenerated in situ to enable a second read from the opposite end of the fragments. Once the original templates were cleaved and removed, the reverse strands were subjected to sequencing-by-synthesis. Genomic DNA was also fragmented into 100–400 bp segments and subjected to the same library construction and sequencing.

### *De novo* assembly of cDNA

A next-generation variant calling tool was used for SSR discovery [29]. Prior to the assembly, we conducted a stringent filtering process of raw sequencing reads. The reads with > 10 % of bases with a quality score of Q < 20, non-coding RNA (such as rRNA, tRNA, and miRNA) ambiguous sequences represented as “N” and adaptor contamination were removed. *De novo* assembly of transcriptome and genome was performed with Trinity and SOAPdenovo2, respectively, using the *de Bruijn* graph method and default settings except for the K-mer value. The k-mer value with the best N50 size was selected for final assembly.

### Development and detection of SSR markers

The MISA (http://pgrc.ipk-gatersleben.de/misa/) script was used to identify microsatellites in the unigenes and assembled genome contigs. The software was used to design PCR primers. Forward and reverse SSR primer pairs based on the flanking sequences of the SSR loci were designed by running the software in batch mode. The primers varied in length from 18 to 20 bp (the optimal length: 20 bp), with GC contents varying between 45 % and 65 % (optimal GC content: 50 %). These preliminary primer pair sequences were validated by *in silico* PCR analysis against the *B. rapa* genome and all possible amplifications were determined using the BLASTN program. Genome sequences of these three species were download from the public database (*Brassica rapa*: http://www.ncbi.nlm.nih.gov/genome/229; *Brassica oleracea*: http://www.ncbi.nlm.nih.gov/genome/10901; *Brassica napus*: http://www.ncbi.nlm.nih.gov/genome/?term=brassica%20napus).

PCR amplification was conducted in the following conditions: DNA was denatured at 94 °C for 4 min; followed by 35–40 cycles of 94 °C for 30 s, 55 °C–60 °C for 30 s, and 72 °C for 2 min; and a final extension at 72 °C for 10 min. The PCR products were analyzed by electrophoresis on 8.0 % non-denaturing polyacrylamide gels with ethidium bromide. The band sizes were determined against a DNA ladder. A total of 19 EST-SSR primers pair and 213 genomic SSR primer-pairs were used in the experiment. The amounts of each primer pair were based on the ratio between the detected EST-SSRs and genomic SSRs.

### Data analysis

The PIC value was calculated using the formula (PIC = 1-∑ (Pi)^2^), where Pi is the proportion of samples carrying the allele of a particular locus. The genetic distances were calculated with the NTSYSpc (version 2.1 s) software (http://www.exetersoftware.com/cat/ntsyspc/ntsyspc.htmlhttp://www.exetersoftware.com/cat/ntsyspc/ntsyspc.html) with the coefficient of NEI72. In addition, UPGMA was adopted for cluster analysis and to generate a representative tree plot. By assuming Hardy–Weinberg disequilibrium, the POPGENE software (version 1.31; https://www.ualberta.ca/~fyeh/popgene_download.html) was used to calculate the Gst and Nm using the formula: Nm = 0.5 (1 - Gst)/Gst.

## Abbreviations

EST, expressed sequence tag; NGS, next-generation sequencing; PIC, polymorphism information content; RAPD, random amplified polymorphic DNA; SNP, single nucleotide polymorphisms; SSR, simple sequence repeat; UPGMA, unweighted pair group method analysis

## Additional files

Additional file 1: The total dataset of the EST-SSR primer sequence. (XLSX 172 kb) Additional file 2: The total dataset of the genomic-SSR primer sequence. (XLSX 3479 kb) Additional file 3: Primers mapped on *Brassica rapa* genome (XLSX 177 kb) Additional file 4: Primers mapped on *Brassica oleracea* genome (XLSX 174 kb) Additional file 5: Primer mapped on *Brassica napus* genome (XLSX 101 kb) Additional file 6: The 166 primer-pairs validated by PCR. (XLSX 25 kb) Additional file 7: The genetic distances with the coefficient of NEI72 among accessions. (XLSX 14 kb)
